# An insight into non-emissive excited states in conjugated polymers

**DOI:** 10.1038/ncomms9246

**Published:** 2015-09-22

**Authors:** Zhongjian Hu, Adam P. Willard, Robert J. Ono, Christopher W. Bielawski, Peter J. Rossky, David A. Vanden Bout

**Affiliations:** 1 Center for Nano- and Molecular Science and Technology, Department of Chemistry, University of Texas at Austin, Austin, Texas 78712, USA

## Abstract

Conjugated polymers in the solid state usually exhibit low fluorescence quantum yields, which limit their applications in many areas such as light-emitting diodes. Despite considerable research efforts, the underlying mechanism still remains controversial and elusive. Here, the nature and properties of excited states in the archetypal polythiophene are investigated via aggregates suspended in solvents with different dielectric constants (*ɛ*). In relatively polar solvents (*ɛ*>∼ 3), the aggregates exhibit a low fluorescence quantum yield (QY) of 2–5%, similar to bulk films, however, in relatively nonpolar solvents (*ɛ*<∼ 3) they demonstrate much higher fluorescence QY up to 20–30%. A series of mixed quantum-classical atomistic simulations illustrate that dielectric induced stabilization of nonradiative charge-transfer (CT) type states can lead to similar drastic reduction in fluorescence QY as seen experimentally. Fluorescence lifetime measurement reveals that the CT-type states exist as a competitive channel of the formation of emissive exciton-type states.

## Abstract (web summary)

Conjugated polymers in thin films exhibit low fluorescence quantum yields, but the mechanism is still unclear. Here, Hu *et al*. show the trade-off between charge transfer and emissive exciton states, whilst the former can be suppressed via dielectric-induced stabilization for large fluorescence quantum yields.

## Graphical abstract

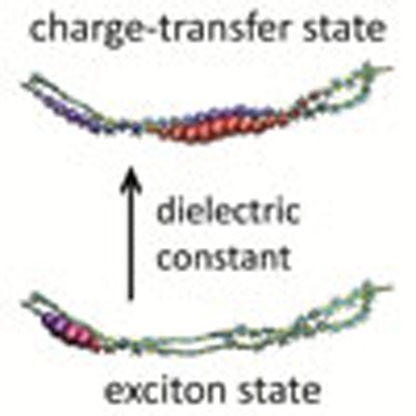

Polythiophene and its derivatives have been extensively utilized in various organic electronic devices including solar cells^1^,^2^, field-effect transistors^3^ and light-emitting diodes^4^. Structurally, the prototypical regioregular poly(3-hexylthiophene) (rr-P3HT) usually forms two dimensional lamellae structures as a result of close interchain packing interaction^5^. Upon going from isolated polymer chains to closely packed bulk states, the optical and electronic properties alter significantly due to electronic interaction between polymer chains. The highly ordered interchain packing not only favors long-range delocalization of excited state wave functions, but also can efficiently funnel the energy down to low energy sites where polymer chains have long conjugation, making the emission dominated by the local energetic minima. Undoubtedly, the nature and character of excited states in the presence of interchain interactions highly affects important photophysical properties of conjugated polymers (CPs). Empirically, the interchain electronic interaction leads to a dramatic decrease in fluorescence efficiency that strongly limits the application of CPs as emissive materials such as in light emitting diodes^4^. The delocalization, dissociation, and recombination of excited states due to interchain interaction are also of keen interest in bulk heterojunction solar cells. Hence a fundamental understanding about the properties of excited states is of vital importance in realizing desired function and performance in a number of applications for CPs.

Many research efforts have been dedicated to account for the dramatic decrease in fluorescence quantum yield (QY) of P3HT in the presence of interchain interaction. A weakly-interacting H-aggregate model, in which the 0-0 transition from the first excited state to the ground state is symmetry forbidden, has been developed by Spano *et al*.^6^ Singlet-triplet exciton annihilation^7^, polarons, and polaron pairs^8^,^9^,^10^ have also been demonstrated to be partially responsible for the loss of fluorescence. Moreover, Silva et al. reported a significant charge-transfer (CT) character of excitons through delayed photoluminescence spectroscopy and quantum-chemical calculations^11^. Recently, Scheblykin et al reported a branching between the formation of emissive excitons and dark states that exist as either static or ultrafast deactivation processes^12^. Despite these research findings, the real nature and character of excited states of P3HT due to the interchain interaction is still not fully appreciated. The problem mainly arises from the inherent structural heterogeneity in commonly investigated bulk films, where various interchain and intrachain morphologies coexist, and a relatively limited number of physical parameters exist in films to tune. Hence, a novel material system is imperative for elucidating the in-depth excited state photophysics and therefore providing potential channels for tuning properties and functions of CPs.

Molecular aggregates suspended in solution have been demonstrated as an excellent model in interrogating the structure-property relations of CPs due to relatively simplified morphological heterogeneity compared to the bulk^13^,^14^,^15^,^16^,^17^,^18^. More importantly, this unique material system offers an opportunity in probing the fundamental characteristics of excited states by altering the solvent dielectric constant (*ɛ*). In our previous work, the idea about the excitonic and CT characters in excited states were tentatively discussed by comparing the fluorescence quantum yield of polythiophene aggregates in only two solvent mixtures^13^. However, a systematic study (i.e., a broad range of dielectric rather than two data points at two extremes of dielectric) to probe the nature and character of excited states (i.e., theoretical calculation and formation mechanism) is lacking. In present work, P3HT aggregates with film-like absorption/emission spectra are fabricated using a triblock copolymer of P3HT-b-poly(tert-butylacrylate)-b-P3HT (P3HT-b-PtBA-b-P3HT) (Fig. 1) in a series of organic solvent mixtures with different dielectric constant *ɛ* (i.e., from polar to non-polar) to quantitatively examine the effect of *ɛ* on the excited state. The excitonic and CT characters of excited states of the aggregates are envisioned based on experimental result of fluorescence QY as a function of solvent *ɛ* and then interrogated with quantum-classical atomistic simulations. Furthermore, a fluorescence lifetime experiment illustrates a branching formation mechanism of the CT-type and exciton-type states.

## Results

### Mimic P3HT film spectra in solvent mixtures

**Figure 2:**
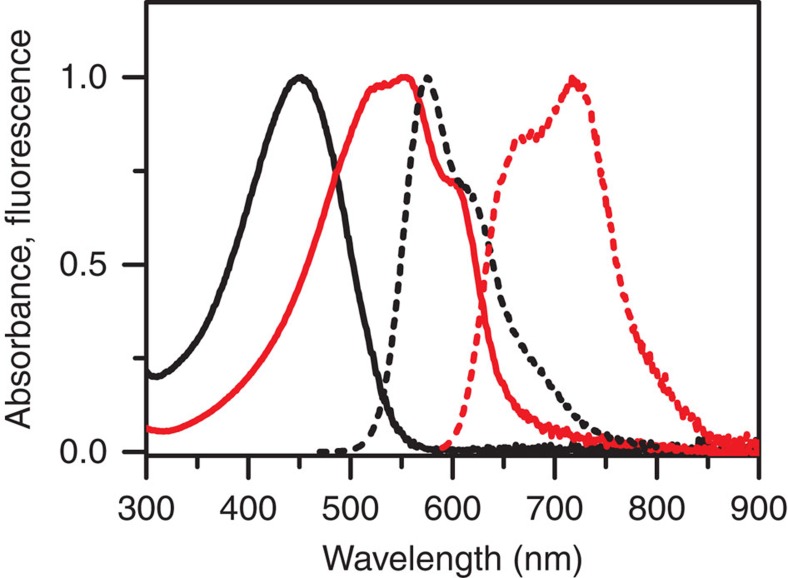
Spectra of P3HT homopolymer. Normalized absorption (solid) and fluorescence (dashed) spectra of homopolymer P3HT (10 kDa) toluene solution (black) and bulk film (red). The fluorescence spectra were taken under excitation at the wavelength with maximum absorbance.

Figure 2 displays typical absorption and fluorescence spectra of a molecular solution and a bulk film of rr-P3HT homopolymer (number average molecular weight (*M*_n_) = 10 kDa; dispersity (*Ð*) = 1.2) spin cast from toluene solution. As shown, the solution has a broad absorption band peaked at 450 nm. The absorption spectrum of bulk film exhibits a red shift with an absorption maximum at 560 nm and pronounced vibronic structures at 525 and 610 nm, which are attributed to increased planarity of P3HT backbones and coupling between C=C stretching and electronic transition^19^. With respect to fluorescence, a strong red shift is also observed for the P3HT film relative to the molecular solution due to enhanced polymer backbone planarity and efficient energy migration to low energy sites. There is also a dramatic decrease in the fluorescence QY of bulk film relative to the molecular solution, which has been ascribed to aforementioned phenomena. To mimic the spectral characteristics of bulk P3HT films, molecular aggregates suspended in solution have proven to be a simplified and effective model. It has been demonstrated previously that in solution, aggregation of P3HT can be initiated by adding a poor solvent for P3HT such as methanol into molecular solutions in a good solvent such as toluene or by lowering the temperature^13^,^14^,^15^,^16^,^17^,^18^. As a further step of our previous investigation of using the triblock copolymer P3HT-b-PtBA-b-P3HT in solution to mimic the P3HT bulk film properties^13^, herein, the aggregation of P3HT was accomplished in solvent mixtures of toluene and a series of poor solvents. Compared to P3HT hompolymer, the close proximity between two P3HT segments in one single triblock molecule promotes better aggregation of P3HT in poor solvents (Supplementary Fig. 1). In addition, the triblock offers stable aggregates that remain suspended in solution for long periods of time^13^. Investigating molecular aggregates in solution allows the electronic states due to interchain interaction to be probed as a function of solvent dielectric. The poor solvents range from highly polar acetonitrile and methanol to relatively nonpolar 1,4-dioxane and hexane, and are listed in Table 1 with their dielectric constants and usage in preparing aggregates.

### Aggregation behavior of triblock in solvent mixtures

When triblock molecules are transferred from toluene to toluene/poor solvent media, one question arises as to whether triblock chains collapse individually (i.e., single polymer chain aggregate) or pack together to form aggregates (i.e., aggregates composed of multiple polymer chains). Typically this question can be simply addressed by examining the absorption and emission spectra of P3HT. However, the triblock copolymer P3HT-b-PtBA-b-P3HT in present work has two P3HT segments, therefore one still cannot determine if the aggregate-like spectra (both for absorption and emission) are from collapsed single triblock molecules or ‘big' aggregates of multiple triblock molecules. To answer this question, we applied fluorescence correlation spectroscopy (FCS) to examine the variation in number of emitters upon going from toluene to toluene/poor solvent mixture. The mean number of emitters in the FCS observation volume can be obtained from the correlation function amplitude of FCS curves, which can be extracted from fitting the curves using correlation function equation (see Supplementary Fig. 2 for examples of fits)^20^. However, it should be noted that FCS analysis is susceptible to background fluorescence (e.g., detector dark counts, impure solvent, scattered light, etc.)^21^,^22^. The uncorrelated background signal lowers correlation function amplitude and hence leads to the concentration of emitters being overestimated. This effect becomes dramatic when fluorescence intensity is not significantly above the background signal, such as the case of the triblock in polar solvent media in this study. In the presence of such background, the measured correlation function amplitude should be corrected by a factor of <F_(t)_>^2^/[<F_(t)_>−<F_BG_>]^2^, where <F_(t)_> and <F_BG_> are the time-averaged total fluorescence signal and background signal, respectively (Supplementary Note 1 and Supplementary Fig. 2)^22^. Furthermore, it should be noted that FCS measurement is based on the statistical analysis of fluorescence intensity fluctuation and is not affected by the fluorescence intensity (i.e., quantum yield) of different samples as long as the emitters can be detected in FCS.

**Figure 3:**
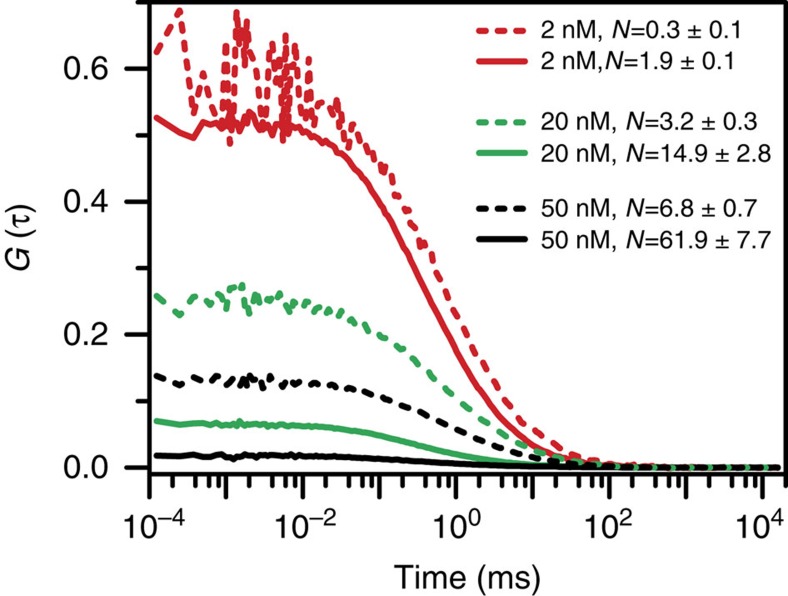
FCS correlation data. FCS correlation curves for triblock in toluene (solid lines) and toluene/methanol (dashed lines) at three same initial triblock concentrations. The inset presents the concentration and the number of emitters after background correction.

Figure 3 displays FCS curves for triblock in toluene and toluene/methanol (50/50 vol.%) mixture at different concentrations with the number of emitters obtained after background correction. As can be seen, for the triblock in toluene, the measured number of molecules approximately scales up as expected with concentration. Although the correlation function amplitude of 2 nM triblock in toluene/methanol (50/50 vol.%) seems overlapped with that of 2 nM triblock toluene molecular solution, after background correction the real number of emitters of the former drops ∼ 6 times relative to the latter. As shown in Fig. 3, with same initial triblock concentrations, when going from toluene to toluene/methanol, the number of emitters approximately drops 5–10 times. This result reveals that triblock forms aggregates of ∼ 5–10 triblock molecules in the toluene/methanol solvent mixture. In addition, we have tested different excitation powers in the FCS measurement and did not find detectable variation in the number of emitters, indicating that there is no highly heterogeneous distribution of fluorescence efficiency of the aggregates. Similar to the triblock in toluene/methanol, aggregation behavior of ∼ 5–10 polymer chains has also been observed for triblock in other solvent mixtures such as toluene/hexane (Supplementary Fig. 3). These data imply that there is no obvious variation in the number of aggregates in different solvent mixtures. Furthermore, the small detection volume in FCS experiment limits the number of emitters observed to be only a few statistically especially for low concentration samples. We observe in FCS experiments that the mean photon count rates (i.e., intensity) of triblock aggregates in toluene/methanol is about 10 times less than that of aggregates in toluene/hexane, suggesting that statistically individual aggregates in toluene/methanol are approximately ∼ 10 times less bright than those in toluene/hexane. This observation corresponds with bulk sample data that the aggregates exhibit a low fluorescence QY of ∼ 2% in toluene/methanol and QY of up to ∼ 30% in toluene/hexane (see data hereinafter). Based on these above results, we do not think there are a significant number of non-emissive aggregates for triblock in bad solvents even for toluene/methanol mixture.

In addition, the fitting results (Supplementary Fig. 4) for FCS curves in Fig. 3 showed that the diffusion time (*τ*_D_) of triblock in solvent mixture is about 3 times longer in general (i.e., diffusion coefficient (*D*) is about 3 times smaller) relative to triblock in toluene. This data suggests larger hydrodynamic radii and hence more aggregation of triblock in solvent mixture than in toluene. It also implies that the drop in the number of emitters observed above (Fig. 3) for triblock from good solvent toluene to poor solvent mixtures is not due to the generation of non-emissive triblock single molecules (i.e., single chains merely collapse and don't aggregate). Otherwise, the diffusion time measured in the FCS experiment would be the same (or even faster as the hydrodynamic radius of the collapsed triblock would be smaller). Furthermore, our recent coarse-grained simulation did reveal the formation of cylindrical-shaped triblock aggregates^23^. Collectively, our data clearly reveals that the film-like spectral features observed for the triblock in toluene/poor solvent mixtures arises from aggregates comprising several triblock molecules rather than collapsed individual triblock chains with two P3HT ends packed together.

### Absorption and fluorescence spectra

**Figure 4:**
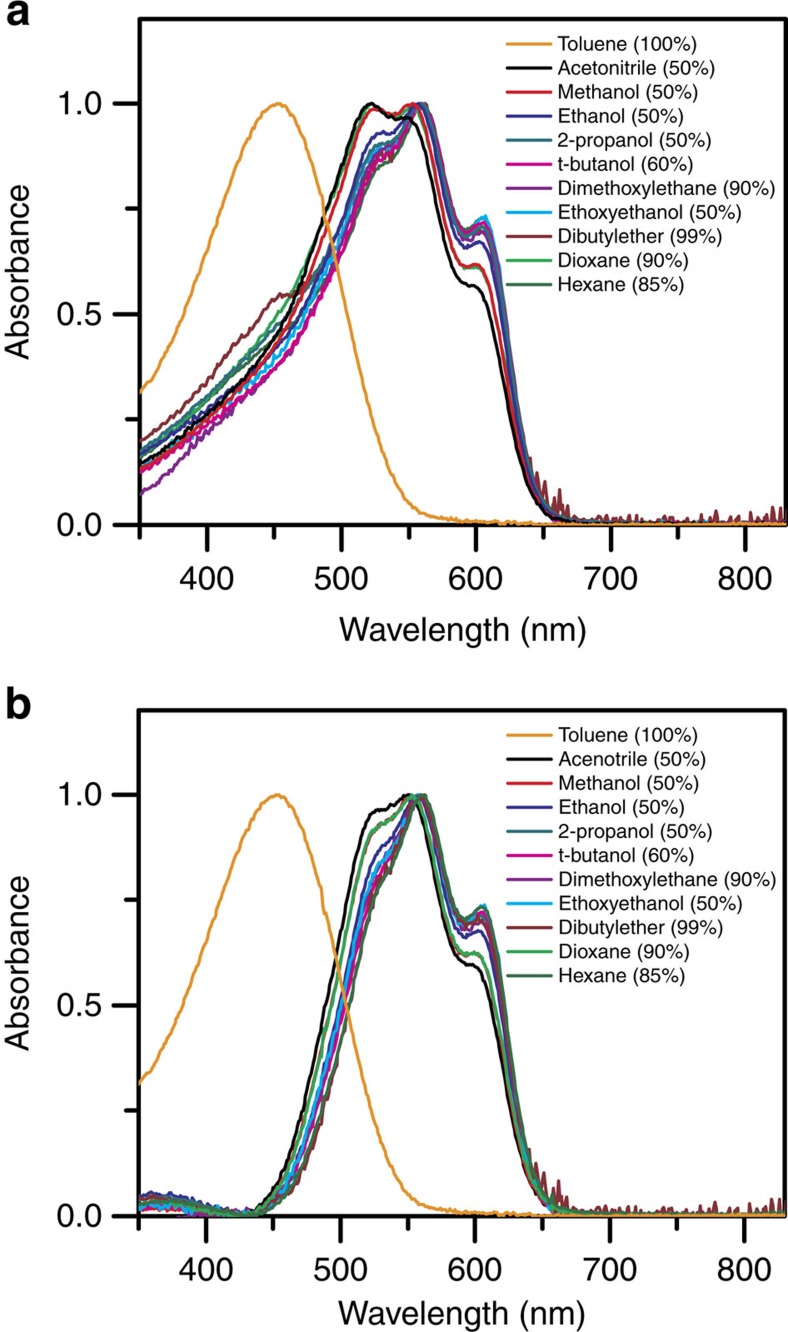
Absorption spectra. (**a**) Normalized absorption spectra of triblock in different mixtures of toluene and poor solvents (normalized to the maximum absorbance). The volume percent of poor solvent in each mixture are shown in parentheses. (**b**) Extracted absorption spectra of pure aggregates for each solvent mixture (normalized to the 0-1 transition peak). The absorption spectra of triblock molecular solution in toluene are included in (**a**) and (**b**) for comparison.

Similar to what we observed previously, with increasing amount of poor solvent in toluene/poor solvent mixtures, red shifted spectra with an emergence and gradual intensity increase of vibronic structures at 560 and 610 nm due to interchain interaction were observed^24^. Eventually, the spectral change saturates at a certain volume ratio of toluene/poor solvent (Table 1) at which point there is a maximum fraction of aggregates contributing to the absorption spectrum. The final absorption spectra of triblock in different solvent mixtures are shown in Fig. 4a with volume percentage of poor solvents in the parentheses. The triblock absorption spectra in the solvent mixtures, however, contain contributions from both aggregated and molecular triblock chains^13^,^14^. To extract pure spectra of aggregates, the molecular solution spectrum of triblock in toluene was scaled to the low wavelength shoulder of the spectra in solvent mixtures first and then was subtracted^14^. The extracted spectra of aggregates in different solvent media, as shown in Fig. 4b, are similar with slight differences in the relative intensity of vibronic transitions. A close examination reveals that the 0-0 electronic transition gradually increases with decreasing dielectric constant from toluene/acetonitrile to toluene/t-butanol, while it is similar for all of the low dielectric solvent mixtures except for toluene/dioxane. Within the framework of weak interchain coupling in the H-aggregate model, the interchain excitonic coupling (*J*_0_) can be estimated from the ratio of peak absorbance of *A*_0-0_ and *A*_0-1_, for which increased ratio implies reduced interchain coupling (Supplementary Fig. 5)^25^. For excitonic coupling, the screening factor by solvent medium is a function of optical dielectric constant (*ɛ*_opt_), which is equivalent to the square of refraction index (*n*)^26^,^27^. Since the *n* values of the solvent mixtures studied herein are very close, i.e., in the range of 1.37–1.45, the change in interchain coupling caused by the variation of solvents is anticipated to be small. We believe that the slight difference in interchain excitonic coupling for the aggregates studied in different solvent mixtures is mostly due to a subtle change in packing morphology of polymer chains. In addition, the well dissolved side-chains in nonpolar solvent environments would also be expected to reduce torsional disorder of P3HT backbone, therefore benefitting long conjugation length and high ordering along P3HT chain. The increased intrachain order, in turn, can also lead to a decrease in interchain excitonic coupling^28^,^29^,^30^,^31^.

**Figure 5:**
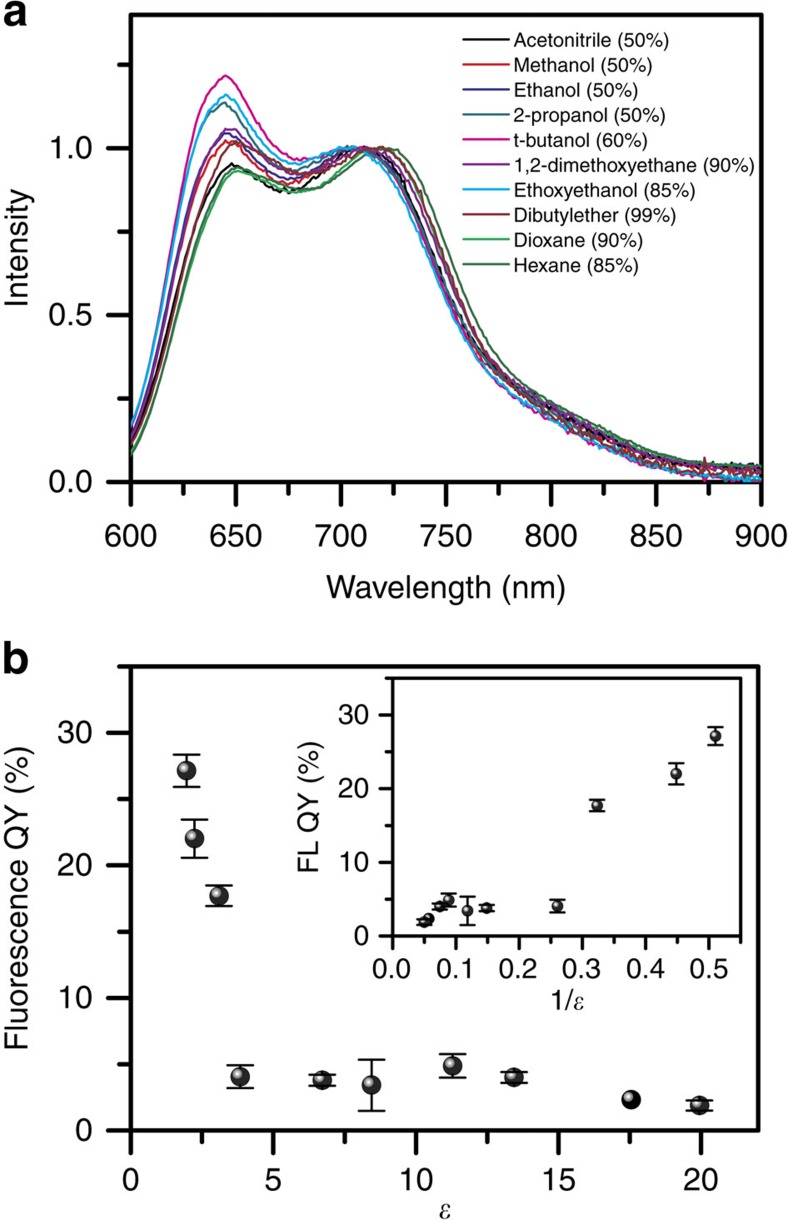
Fluorescence spectra and quantum yield. (**a**) Fluorescence spectra (normalized to the 0-1 transition peak) of the triblock in solvent mixtures of toluene and poor solvents under excitation at 560 nm. The volume percent of poor solvent in each mixture is shown in the parenthesis. (**b**) Fluorescence quantum yield (QY) of the triblock as a function of dielectric constant (*ɛ*) of the solvent mixtures. From left to right, the solvent mixture becomes more and more polar as listed in Table 1. The inset presents the plot of QY versus the reciprocal of dielectric constant. From left to right, the solvent mixture becomes more and more nonpolar. The error bars in (**b**) and inset depict the standard deviations of the fluorescence QY.

Figure 5a shows fluorescence spectra of triblock aggregates in solvent mixtures under excitation at 560 nm, at which only aggregated P3HT can be excited. These spectra exhibit bulk film-like spectral profile but with slight variation in vibronic structure. As the poor solvent evolves from acetonitrile to t-butanol, the intensity of 0-0 transition gradually increases relative to the 0-1 transition. However, from 1,2-dimethoxyethane to hexane, the *I*_0-0_/*I*_0-1_ ratio generally drops without correlation with the *A*_0-0_/*A*_0-1_ ratio (i.e., the coupling strength in Supplementary Fig. 5). For aggregates showing high *A*_0-0_/*A*_0-1_ratio, a high *I*_0-0_/*I*_0-1_ ratio would also be expected according to the weakly coupled H-aggregate model or even the HJ aggregate model^6^,^28^. However, in these models, the 0-0 emission intensity is highly susceptible to energetic disorder^11^,^28^. In addition, the emission spectral profile is also strongly affected by ultrafast vibrational relaxation (i.e., torsional planarization along P3HT backbone) and energy migration upon photoexcitation^11^,^32^. These factors lead to the analysis concerning electronic coupling based on vibronic structures of emission to be relatively less informative and complicated than based on absorption characteristics.

### Fluorescence quantum yield

While the fluorescence spectra of the aggregates show only slight variation in different solvent mixtures, the fluorescence QY of the aggregates exhibits large variations. In particular, there is a systematic variation in the yield for emission as a function of the dielectric constant *ɛ* of the solvent. Since the polymers are in a solvent mixture, the dielectric is taken as the volume fraction weighted average of the dielectrics of the individual pure solvents. Figure 5b shows the fluorescence QY as a function of dielectric. In the solvent mixtures with *ɛ* above ∼ 3 (i.e., from the mixture of toluene/acetonitrile (*ɛ*=19.94) to toluene/dimethoxyethane (*ɛ*=6.72) and toluene/ethoxyethanol (*ɛ*=3.84)), the fluorescence QY of triblock aggregates remains low in the range of 2–5%. This is similar to the value typically observed for the QY of bulk P3HT films. In contrast, when *ɛ* is below 3 (i.e., for toluene/hexane, toluene/1,4-dioxane, and toluene/dibutylether) the QY increases dramatically up to values in the range of ∼ 20–30%. This is nearly an order of magnitude higher than that of the aggregates in the high dielectric solvents, and close to typical QY of 30–40% of P3HT molecular solution^8^,^13^,^33^.

A close interchain packing and efficient π electron overlap generally would allow for the formation of CT-type states in conjugated polymers^34^,^35^. The existence of CT-type states has been suggested in films for the prototypical conjugated polymer polythiophene^19^,^36^,^37^,^38^. Since the oscillator strength of CT-type states is generally small as a result of limited overlap between electron and hole wave functions, the electronic transition of CT excitons is hardly detected in typical absorption and emission spectra. Due to the larger dipole moment of CT states relative to the exciton-type states, its energies respond more sensitively to changes in dielectric, that is, the relative energies of CT-type and exciton-type states depend on the solvent dielectric. Considering the fluorescence is dominated by the lowest energy excited state (Kasha's rule), the possible dielectric-induced crossing of CT-type and exciton-type states would result in a precipitous change in fluorescence QY. As described above, when the solvent dielectric is changed from *ɛ* ≥ 4 to *ɛ* ≤ 3, the triblock aggregates exhibit a steep change in fluorescence QY. We think that these observations signify the existence of CT character and exciton character in the aggregate excited state. In the solvent mixtures with high *ɛ*, the poorly emissive CT-type states are energetically stabilized and the lowest energy states would be primarily those with CT character and low emission yield. In contrast, in the solvent mixtures with low *ɛ*, CT-type states are raised in energy relative to their highly emissive exciton-type counter parts, therefore leading to high fluorescence QY. For bulk P3HT films, it has been estimated that ɛ lies in the range of 3.0–7.0 (refs 39, 40, 41, 42, 43), which locates the bulk film in the relatively high ɛ range. Therefore, the low fluorescence QY in P3HT films should be at least partially attributed to nonradiative CT-type states. Such states in bulk film have been probed by Paquin et al. using time-resolved spectroscopy and quantum-chemical calculations. The authors found that the CT-type states mostly occur at the interface between ordered and disordered domains, driven possibly by energetic disorder^11^,^36^.

Besides the effect of solvent dielectric, variations in intermolecular packing structure in different solvent mixtures might also modify the exciton and CT character for stacked chromophores. Several recent theoretical works have indicated the CT character in excited state is strongly affected by the longitudinal and lateral displacement between adjacent stacked chromophores^37^,^44^,^45^. According to Spano's calculations on stacked oligothiophenes^37^, the lateral or longitudinal translations between the chains would change the coupling between CT and Frenkel excitons, which would lead to variation in the character of aggregate, i.e., H- and J-aggregate. As can be seen from the absorption and emission spectra of triblock aggregates in solvent mixtures (Fig. 4b and Fig. 5a), there is only slight change in the vibronic structure between each other, which might be due to slight difference in packing structure. However, we did not observe significant or gradual changes in electronic transition energy and vibronic structure for the spectra of triblock aggregates in different solvent mixtures. Therefore, we think that the influence on excited state characters by a possible translational shift between adjacent P3HT chains (resulting from different solvent mixtures) is probably insignificant in present case.

### Fluorescence lifetime

**Figure 6:**
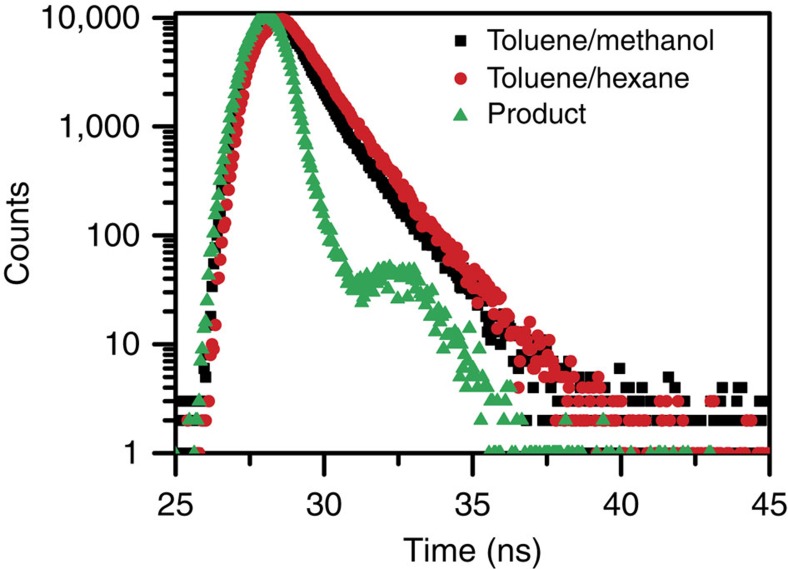
Fluorescence lifetime. Fluorescence decay dynamics at 720 nm of P3HT triblock in high dielectric (toluene/methanol=50/50 vol.%) and low dielectric (toluene/hexane=15/85 vol.%) solvent mixtures. The instrument response function (prompt) is obtained at 639 nm (excitation wavelength) using Ludox suspension.

The fluorescence lifetime for the triblock in pure toluene, which is ∼ 570 ps, similar to the lifetime of typical polyalkylthiophenes in solution^8^,^17^,^33^,^46^. To obtain a further understanding about the CT and excitonic character in the excited states of aggregates, we carried out fluorescence lifetime measurement for triblock aggregates in different solvent mixtures. Figure 6 presents the fluorescence decay profiles of aggregates in high and low dielectric solvent mixtures (i.e., toluene/methanol and toluene/hexane as a representative, respectively). As shown, the emission decays of aggregates in both solvent mixtures are approximately overlapped with an average decay time of ∼ 700 ps, similar to literature results^9^,^46^.

The proximity of the lifetime of the emissive excitonic state of the triblock in both polar and nonpolar solvents is somewhat unexpected, since a much shorter lifetime would be expected for the aggregates with such low fluorescence QY in high dielectric media. Considering that the triblock aggregates in different solvent mixtures have a similar value of absorption cross-section, the radiative decay rates of the aggregates should be fairly similar. These two observations indicate that the CT-type state as a nonradiative deexcitation channel in high dielectric environment forms not via the exciton-type state. Scheblykin et al recently proposed a branching formation scheme of the emissive exciton-type states and the dark states^12^. The latter could be a result of either ultrafast deactivation or static quenching, although the unresolved ultrafast quenching might appear as static quenching. We think that there exists a branching between the generation of exciton-type and CT-type states (i.e., dark states). On one hand, upon photoexcitation, the primary excitation species in conjugated polymers is vibrationally ‘hot' singlet exciton, as documented by ultrafast spectroscopic studies^47^,^48^,^49^. These ‘hot' excitons can relax rapidly through geometrical relaxation (mostly torsional relaxation) to form low energy sites such as relaxed excitonic state, CT state^36^,^50^, or polaron pairs and polarons^8^,^10^,^48^. In this scenario, the branching fraction of exciton-type state strongly relies on the relative energies of CT-type and exciton-type states, which, in turn, are dominated by the solvent dielectric property in our case. On the other hand, for the static quenching scheme^12^,^51^, it might also be possible that the CT-states result from so far unknown complexes. And, herein, the formation yield of these complexes or CT-states might have a dependence on solvent dielectric.

Nonetheless, the observation of ‘direct' generation of CT-type states in triblock aggregates substantiates previous studies about the generation of photoexcitations in bulk P3HT state. The branching between polaron-pairs (or polarons) and exciton has also been reported via transient absorption spectroscopy for P3HT films^8^,^48^, where long range photoexcitation delocalization, material morphology and resultant disordered energetic landscape would work cooperatively in transferring CT-type state into polaron-pairs or polarons.

### Quantum-classical atomistic simulations

To evaluate the validity of a screening-induced stabilization of low-lying CT-type states, we have carried out a series of hybrid quantum-classical atomistic simulations of two pi-stacked thiophene 30-mers embedded in a tunable dielectric environment. The approximation made here, of a single constant dielectric constant equal to that of the solvent, is equivalent to, first, assuming that charges on the polymers are screened as well as they would be in the bulk solvent and, second, to assuming that the solvation effect is correctly treated as a featureless dielectric continuum. Of course, neither of these can be quite right, since the polymer electronic structure will change when charges are present, so that a dielectric constant is simplistic, and the solvent is a molecular material whose granularity is relevant at the small length scales present here. However, the general trends one sees in a continuum dielectric screening model should be of the same form as one would see if these aspects were treated more accurately. On the other hand, the fact that the polymer is itself composed of groups that are associated with low dielectric constant materials does not imply that the screening of charges should be poor^52^,^53^. The dielectric screening is not a property of the nearby material alone, but involves non-local effects associated with the fact that a high dielectric material largely surrounds the charges at larger distances. Hence, as has been seen in other contexts^54^, unless the charges are well buried (∼ > 1 nm) in a non-polar environment that is secluded from the solvent, the apparent charge-charge interaction can still be quite effectively screened by the surrounding solvent. Specifically the system was modeled following the QCFF/PI method of Warshel and Karplus^55^ in which the molecular system is partitioned into two subsystems, one containing explicit quantum mechanical detail and the other in which such details are accounted for implicitly. In our case the quantum subsystem contains only the pi-electrons which are described using a semi-empirical Pariser-Parr-Pople (PPP) Hamiltonian^56^,^57^,^58^ and all remaining degrees of freedom (nuclear as well as the core and sigma electrons) belong to the classical subsystem which is modeled as a positively charged nuclear scaffold that evolves via a molecular mechanics force field. This simulation methodology has recently been applied to a variety of similar systems^59^,^60^,^61^,^62^,^63^. Further details of the model can be found in the Supplementary Method 1 and Supplementary Table 1 and 2.

**Figure 7:**
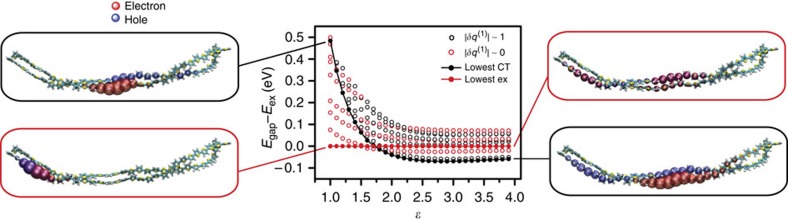
Dielectric-induced stabilization of the charge transfer state. The graph (center) indicates the energy levels vs. dielectric constant of the ten lowest excited states, plotted relative to the lowest exciton-type state, for a single ground state configuration of the dimer system. Points are colored according to *δq*^(1)^, the excess charge on one molecule. The lowest energy CT-type and exciton-type states are plotted with solid points. The panels on the left and right contain atomistic renderings of the system at the indicated value of the dielectric constant. The spatial distribution of excess electron and hole represented with semi-transparent red and blue spheres.

Calculations are carried out on an ensemble of 2000 individual configurations harvested from an equilibrium ground state ensemble at T = 298 K. A single calculation consists of a ground state electronic structure calculation followed by the computation of the excited state energy levels via configuration interaction with single excitations (CIS). The Coulombic interactions in the system Hamiltonian are screened through the presence of a uniform dielectric, the value of which is tuned to mimic the effect of different solvent environments. The output of the CIS calculation allows for the straightforward evaluation of the spatial distribution of the excited electron and hole wave functions, which we use to characterize individual excited states as being either exciton-type or CT-type. Specifically we compute *δq*^(1)^, the excess charge due to excitation (in units of electronic charge, *e*) on one of the two molecules. In terms of this excess charge there are two distinct populations of the excited states, those for which |*δq*^(1)^| ≈ 1 and those for which |*δq*^(1)^| ≈ 0. We characterize states belonging to the former population as CT-type states. Similarly, we characterize exciton-type as those with as those with |*δq*^(1)^| = 0 and, in order to exclude polaron-type states, with an electron-hole separation of less than 3.5 Å. A more detailed description of our characterization of excited states is available in Supplementary Note 2 and Supplementary Fig. 6. For individual ground state configurations the low-lying manifold of excited states generally contains a mixture of light-emitting bound exciton states, poorly emitting polaron-type states, and non light-emitting CT states. Since the energy of polar states are more sensitive to changes in dielectric than nonpolar states, the energetic distribution of excited states depends on dielectric constant. Figure 7 shows the effect of dielectric constant on the relative energies of the lowest ten excited states, along with the associated dielectric-induced stabilization of the CT state, for a single ground state configuration. The panels on the left and right contain atomistic renderings of the excited state wave functions at the indicated value of the dielectric constant. The energy of CT states, i.e., those with excess molecular charge |*δq*^(1)^| ≈ 1, are more sensitive to changes in dielectric than for bound states, i.e., those with |*δq*^(1)^| ≈ 0. Among the exciton bound states (colored red in Fig. 7), those that exhibit intermediate sensitivity to ɛ are more polaronic in character.

**Figure 8:**
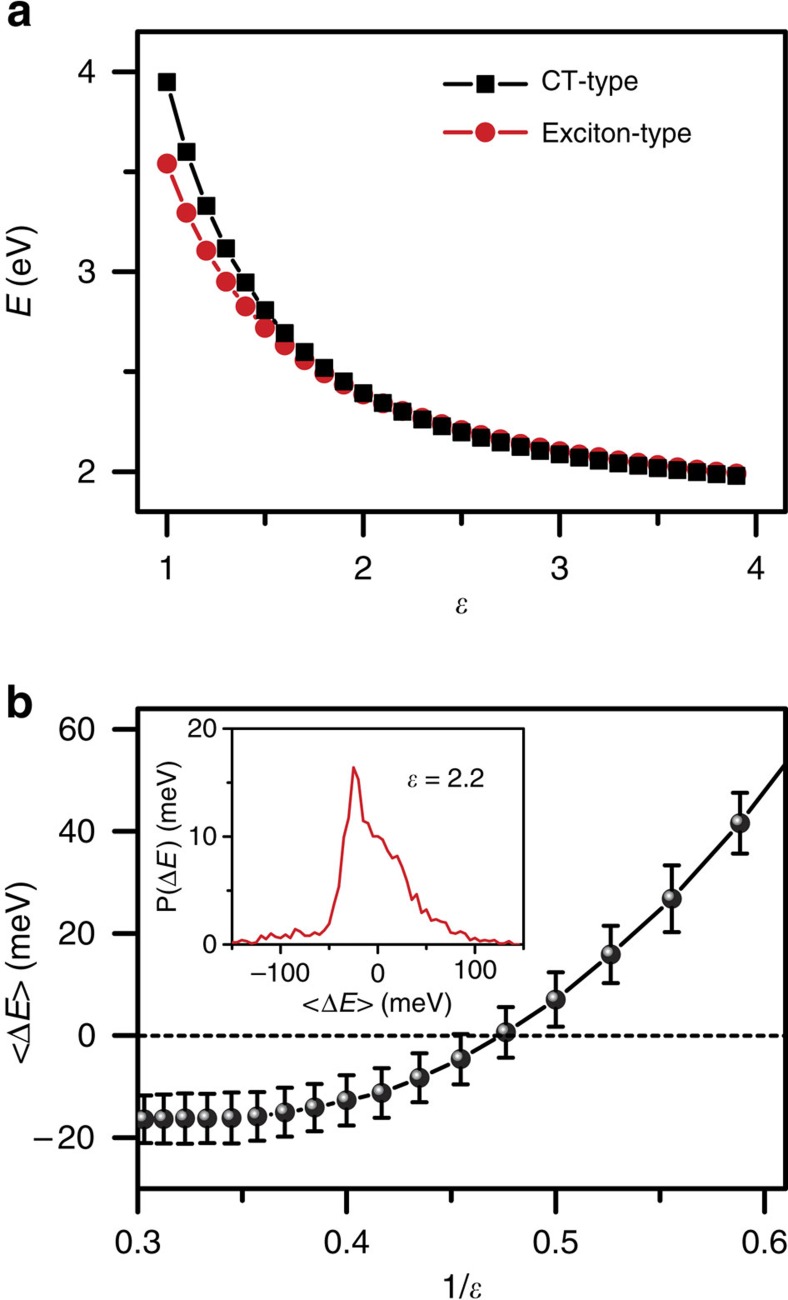
Theoretical calculation of energy levels. (**a**) Simulation results of the dependence of *E*_ex_ (red) and *E*_CT_ (black) on dielectric constant. (**b**) The mean dependence of the Δ*E* = *E*_CT_ − *E*_ex_ on the reciprocal of dielectric constant. Error bars indicate uncertainty associated with finite sample size. Inset in (**b**) shows the equilibrium probability for a configuration to have a given value of Δ*E* at a specific dielectric constant.

For each value of *ɛ* considered, we identified the energies *E*_ex_ and *E*_CT_, the lowest energy excited state with exciton or CT character respectively. Figure 8a shows the average value of *E*_ex_ and *E*_CT_ plotted as a function of *ɛ*, and Fig. 8b presents the average value of Δ*E* = *E*_CT_ − *E*_ex_ versus 1/*ɛ* illustrating the crossover in the identity of the lowest energy excited state. We observe that for *ɛ*<∼2.2 the lowest energy excited state is exciton-type while for *ɛ*>∼2.2 the lowest energy excited state is CT-type. The inferred luminescent consequence of this crossover supports our experimental observation that the fluorescence QY of triblock is much higher in solvent media with low *ɛ*(<∼3) than in media with high *ɛ*(>∼3) (Fig. 5b and inset). The effect of thermal fluctuations on Δ*E* for members of the equilibrium ensemble can be seen in Fig. 8b inset. The quantitative details of these simulation results, for example the value of *ɛ* at which *E*_ex_ and *E*_CT_ cross, depend sensitively on model parameters as well as how exactly solvent-induced screening is implemented within the model (see Supplementary Note 3 and Supplementary Fig. 7). Indeed a more rigorous (and much more computationally intensive) set of calculations would be carried out over an ensemble of relaxed excited-state configurations (rather than ground state) and in the presence of explicit solvent (rather than a uniform dielectric). Nonetheless we expect the qualitative conclusion, that CT-type states can be preferentially stabilized through an enhanced sensitivity to changes in the dielectric screening, will apply generally to this class of systems.

## Discussion

The rr-P3HT film-like spectra of absorption and emission are duplicated with aggregates of a triblock copolymer P3HT-b-PtBA-b-P3HT in a series of solvent mixtures with different dielectric property. The triblock aggregates exhibit a sharp change in fluorescence quantum yield as a function of the dielectric constant *ɛ* of the solvent mixture, i.e., from 2–5% in solvent mixtures with *ɛ*>∼3 to 20–30% in solvent mixtures with *ɛ*<∼3. The experimental result combined with quantum chemical calculations suggests there is a crossover in the energies of CT-type state and exciton-type state as a function of *ɛ*. In low dielectric media the lowest energy excited state is exciton-type. However, in high dielectric media the lowest energy excited state is CT-type, which strongly attenuates the emission efficiency of triblock aggregates. Moreover, the observation of similar fluorescence lifetime of emissive excitons for triblock aggregates in different dielectric solvent media indicates there is a branching between the generation of exciton-type state and CT-type state. That is, the CT-type state exists as a competing channel of the formation of exciton-type state. Our experimental and theoretical results provide a fundamental basis in understanding the basic characteristics of excited states in conjugated polymers. In addition, the discovery of strong dielectric dependence of fluorescence emission efficiency is of practical value for application of conjugated polymers as emissive materials such as in organic light emitting diodes.

## Methods

### Materials

Triblock copolymer P3HT-b-PtBA-b-P3HT was synthesized following the Cu-catalyzed coupling of ethynyl-terminated P3HT with α-diazido-PtBA using a modified, previously reported procedure. A detailed description can be found in our previous report^13^. Pure triblock copolymer was obtained by gel permeation chromatography (GPC, Viscotek, GPCmax VE-2001). The GPC fractionated triblock copolymer has a number average molecular weight (*M*_n_) of 45 kDa and dispersity (*Ð*) of 1.1. The *M*_n_ of P3HT blocks on each side and the PtBA linker is 10 and 25 kDa, respectively. The reference rr-P3HT homopolymer was synthesized using the GRIM method^64^. All the chemical solvents were from Sigma Aldrich or Thermo Fisher Scientific Inc. and used without further purification.

### Sample preparation and optical characterization

The reference rr-P3HT homopolymer (*M*_n_ = 10 kDa, *Ð* = 1.2) film was spin-coated on a microscope cover glass from toluene solution. The triblock was fully dissolved in toluene first, and the poor solvent was added until there was no absorption spectral variation. The UV−vis absorption and fluorescence emission spectra were recorded with a monobeam UV spectrophotometer (Agilent Technologies Inc.) and Fluorolog-3 (Jobin-Yvon) spectrofluorometer, respectively. The comparison of the fluorescence quantum yield for the triblock in different solvent media was conducted using the triblock in toluene/methanol as a reference, which was estimated to be ∼ 2% according to our previous study^13^. To only excite the molecular triblock aggregates in the solvent mixtures, we choose 560 nm as the excitation wavelength. Fluorescence lifetime measurement for the aggregates was taken at 720 nm with a 639 nm pulsed LED laser (Horiba Scientific) with a pulse duration < 200 ps (instrument response function ∼ 300 ps) on the same spectrofluorometer. The instrument response function was obtained at 639 nm using Ludox suspension. Fluorescence correlation spectroscopy was performed on a laser confocal optical microscope (Zeiss Axiovert) with a 1.25 NA objective lens (Zeiss, Achrostigmat, 100 × , oil immersion) operating at 488 nm of an Ar ion laser^13^,^65^,^66^. The fluorescence signal was collected through the same objective, filtered with a 496 nm edge filter, and collected with two avalanche photodiode detectors (SPCM-AQR-15, Perkin Elmer) positioned orthogonally. The detector signals were then correlated by an ALV-5000 fast hardware correlation card to produce FCS correlation trace.

## Additional information

**How to cite this article:** Hu, Z. *et al*. An insight into non-emissive excited states in conjugated polymers. *Nat. Commun.* 6:8246 doi: 10.1038/ncomms9246 (2015).

## Supplementary Material

Supplementary Information Supplementary Figures 1-7, Supplementary Tables 1-2, Supplementary Notes 1-3, Supplementary Method and Supplementary References

## Figures and Tables

**Figure 1:**
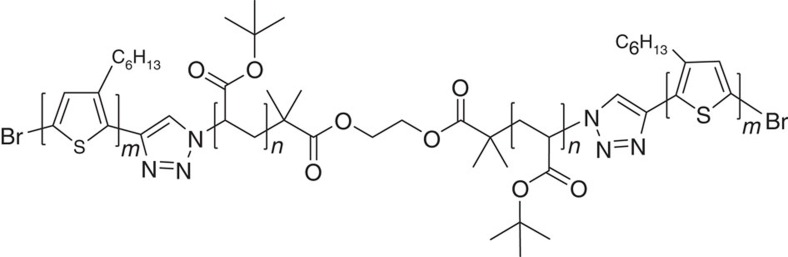
Chemical structure of the triblock copolymer. The triblock copolymer P3HT-b-PtBA-b-P3HT consisting of two P3HT chains covalently linked to both ends of a PtBA chain. The *M*_n_ of P3HT blocks on each side and the PtBA linker are 10 and 25 kDa, respectively. Hence, the values of n and m in the chemical structure are approximately 60 and 100, respectively.

**Table 1:** Organic solvents used in fabricating triblock copolymer aggregates.

**Solvent**	***ɛ*** **(pure solvent)**	**Volume ratio (toluene/poor)**	***ɛ*** **(mixture of toluene/poor)**
Acetonitrile	37.5	50/50	19.9
Methanol	32.7	50/50	17.5
Ethanol	24.5	50/50	13.4
2-propanol	20.2	50/50	11.3
tert-butanol	12.5	40/60	8.5
1,2-dimethoxyethane	7.2	10/90	6.7
Ethoxyethanol	5.3	50/50	3.8
Dibutylether	3.1	1/99	3.1
1,4-dioxane	2.2	10/90	2.2
Hexane	1.9	15/85	2.0
Toluene	2.4	—	—

The dielectric constant ɛ of pure solvent, the volume ratio (toluene/poor) in making aggregates, and the dielectric of mixed solvents (a volume fraction weighted sum of dielectric constants of pure solvents) are listed.

## References

[b1] Li G., Zhu R. & Yang Y. Polymer solar cells. Nat. Photon 6, 153–161 (2012).

[b2] You J. . A polymer tandem solar cell with 10.6% power conversion efficiency. Nat. Comm 4, 4: 1446 (2013). 10.1038/ncomms2411 PMC3660643 23385590

[b3] Nielsen C. B. & McCulloch I. Recent advances in transistor performance of polythiophenes. Prog. Polym. Sci. 38, 2053–2069 (2013).

[b4] Perepichka I. F., Perepichka D. F., Meng H. & Wudl F. Light-emitting polythiophenes. Adv. Mater. 17, 2281–2305 (2005).

[b5] Kim Y. . A strong regioregularity effect in self-organizing conjugated polymer films and high-efficiency polythiophene:fullerene solar cells. Nat. Mater. 5, 197–203 (2006).

[b6] Spano F. C. The Spectral Signatures of Frenkel Polarons in H- and J-Aggregates. Acc. Chem. Res. 43, 429–439 (2010). 20014774 10.1021/ar900233v

[b7] Steiner F., Vogelsang J. & Lupton J. M. Singlet-Triplet Annihilation Limits Exciton Yield in Poly(3-Hexylthiophene). Phys. Rev. Lett. 112, 137402 (2014). 24745453 10.1103/PhysRevLett.112.137402

[b8] Cook S., Furube A. & Katoh R. Analysis of the excited states of regioregular polythiophene P3HT. Energ. Environ. Sci. 1, 294–299 (2008).

[b9] Piris J. . Photogeneration and ultrafast dynamics of excitons and charges in P3HT/PCBM blends. J. Phys. Chem. C 113, 14500–14506 (2009).

[b10] Sheng C. X., Tong M., Singh S. & Vardeny Z. V. Experimental determination of the charge/neutral branching ratio eta in the photoexcitation of pi-conjugated polymers by broadband ultrafast spectroscopy. Phys. Rev. B 75, 085206 (2007).

[b11] Paquin F. . Charge separation in semicrystalline polymeric semiconductors by photoexcitation: is the mechanism intrinsic or extrinsic? Phys. Rev. Lett. 106, 197401 (2011). 21668198 10.1103/PhysRevLett.106.197401

[b12] Sahoo D., Sugiyasu K., Tian Y., Takeuchi M. & Scheblykin I. G. Effect of conjugated backbone protection on intrinsic and light-induced fluorescence quenching in polythiophenes. Chem. Mater. 26, 4867–4875 (2014).

[b13] Brazard J., Ono R. J., Bielawski C. W., Barbara P. F. & Vanden Bout D. A. Mimicking conjugated polymer thin-film photophysics with a well-defined triblock copolymer in solution. J. Phys. Chem. B 117, 4170–4176 (2013). 22296299 10.1021/jp3001256

[b14] Scharsich C. . Control of aggregate formation in poly(3-hexylthiophene) by solvent, molecular weight, and synthetic method. J. Polym. Sci. Pol. Phys 50, 442–453 (2012).

[b15] Millstone J. E. . Synthesis, properties, and electronic applications of size-controlled poly(3-hexylthiophene) nanoparticles. Langmuir 26, 13056–13061 (2010). 20695542 10.1021/la1022938

[b16] Hu Z. & Gesquiere A. J. PCBM concentration dependent morphology of P3HT in composite P3HT/PCBM nanoparticles. Chem. Phys. Lett. 476, 51–55 (2009).

[b17] Rumbles G. . Chromism and luminescence in regioregular poly(3-dodecylthiophene). Synth. Met 76, 47 (1996).

[b18] Shimizu H., Yamada M., Wada R. & Okabe M. Preparation and characterization of water self-dispersible poly(3-hexylthiophene) particles. Polym. J. 40, 33–36 (2008).

[b19] Clark J., Silva C., Friend R. H. & Spano F. C. Role of intermolecular coupling in the photophysics of disordered organic semiconductors: aggregate emission in regioregular polythiophene. Phys. Rev. Lett. 98, 206406 (2007). 17677723 10.1103/PhysRevLett.98.206406

[b20] Lakowicz J. R. Principles of Fluorescence Spectroscopy 3rd edn Springer (2006).

[b21] Lakowicz J. R. Topics in Fluorescence Spectroscopy Vol. 1, Springer (1991).

[b22] Koppel D. E. Statistical accuracy in fluorescence correlation spectroscopy. Phys. Rev. A 10, 1938–1945 (1974).

[b23] Omar A. K. . Aggregation Behavior of Rod–Coil–Rod Triblock Copolymers in a Coil-Selective Solvent. J. Phys. Chem. B 119, 330–337 (2015). 25513935 10.1021/jp509016c

[b24] Bolinger J. C. . Conformation and energy transfer in single conjugated polymers. Acc. Chem. Res. 45, 1992–2001 (2012). 22775295 10.1021/ar300012k

[b25] Spano F. C. Modeling disorder in polymer aggregates: The optical spectroscopy of regioregular poly(3-hexylthiophene) thin films. J. Chem. Phys. 122, 234701 (2005). 16008467 10.1063/1.1914768

[b26] Curutchet C., Scholes G. D., Mennucci B. & Cammi R. How solvent controls electronic energy transfer and light harvesting: Toward a quantum-mechanical description of reaction field and screening effects. J. Phys. Chem. B 111, 13253–13265 (2007). 17973520 10.1021/jp075411h

[b27] Megow J., Renger T. & May V. Mixed quantum-classical description of excitation energy transfer in supramolecular complexes: screening of the excitonic coupling. ChemPhysChem 15, 478–485 (2014). 24470184 10.1002/cphc.201300625

[b28] Yamagata H. & Spano F. C. Interplay between intrachain and interchain interactions in semiconducting polymer assemblies: The HJ-aggregate model. J. Chem. Phys. 136, 184901 (2012). 22583308 10.1063/1.4705272

[b29] Baghgar M., Labastide J. A., Bokel F., Hayward R. C. & Barnes M. D. Effect of polymer chain folding on the transition from H- to J-aggregate behavior in P3HT nanofibers. J. Phys. Chem. C 118, 2229–2235 (2014).

[b30] Niles E. T. . J-aggregate behavior in poly-3-hexylthiophene nanofibers. J. Phys. Chem. Lett. 3, 259–263 (2012).

[b31] Roehling J. D., Arslan I. & Moule A. J. Controlling microstructure in poly(3-hexylthiophene) nanofibers. J. Mater. Chem. 22, 2498–2506 (2012).

[b32] Parkinson P., Muller C., Stingelin N., Johnston M. B. & Herz L. M. Role of Ultrafast Torsional Relaxation in the Emission from Polythiophene Aggregates. J. Phys. Chem. Lett. 1, 2788–2792 (2010).

[b33] Theander M. . Photophysics of substituted polythiophenes. J. Phys. Chem. B 103, 7771–7780 (1999).

[b34] Hadziioannou G. & Malliaras G. G. Semiconducting Polymers, Chemistry, Physics and Engineering Wiley-VCH (2007).

[b35] Scholes G. D. Insights into Excitons Confined to Nanoscale Systems: Electron–Hole Interaction, Binding Energy, and Photodissociation. ACS Nano 2, 523–537 (2008). 19206579 10.1021/nn700179k

[b36] Glowe J.-F. . Charge-transfer excitons in strongly coupled organic semiconductors. Phys. Rev. B 81, 041201 (2010).

[b37] Yamagata H., Pochas C. M. & Spano F. C. Designing J- and H-Aggregates through Wave Function Overlap Engineering: Applications to Poly(3-hexylthiophene). J. Phys. Chem. B 116, 14494–14503 (2012). 23194082 10.1021/jp309407r

[b38] Reid O. G., Pensack R. D., Song Y., Scholes G. D. & Rumbles G. Charge Photogeneration in Neat Conjugated Polymers. Chem. Mater. 26, 561–575 (2014).

[b39] Nolasco J. C. . Extraction of poly (3-hexylthiophene) (P3HT) properties from dark current voltage characteristics in a P3HT/n-crystalline-silicon solar cell. J. Appl. Phys. 107, 044505 (2010).

[b40] Estrada M., Mejia I., Cerdeira A. & Iniguez B. MIS polymeric structures and OTFTs using PMMA on P3HT layers. Solid. State. Electron. 52, 53–59 (2008).

[b41] Cho Y. S. & Franklin R. R. Conducting polymer material characterization using high frequency planar transmission line measurement. Trans. Electr. Electron. Mater 13, 237–240 (2012).

[b42] Mikalo R. P. & Schmeisser D. Electric contacts on conductive polymers: sodium on poly(3-hexylthiophene-2,5-diyl). Synth. Met 127, 273–277 (2002).

[b43] Knipper M. . Impedance spectroscopy on polymer-fullerene solar cells. Z. Naturforsch. A 62, 490–494 (2007).

[b44] Wang L., Olivier Y., Prezhdo O. V. & Beljonne D. Maximizing Singlet Fission by Intermolecular Packing. J. Phys. Chem. Lett. 5, 3345–3353 (2014). 26278443 10.1021/jz5015955

[b45] Crotty A. M. . Molecular Packing in Organic Solar Cell Materials: Insights from the Emission Line Shapes of P3HT/PCBM Polymer Blend Nanoparticles. The Journal of Physical Chemistry C 118, 19975–19984 (2014).

[b46] Ferreira B., da Silva P. F., de Melo J. S. S., Pina J. & Macanita A. Excited-State Dynamics and Self-Organization of Poly(3-hexylthiophene) (P3HT) in Solution and Thin Films. J. Phys. Chem. B 116, 2347–2355 (2012). 22303865 10.1021/jp207418q

[b47] Hwang I. & Scholes G. D. Electronic Energy Transfer and Quantum-Coherence in pi-Conjugated Polymers. Chem. Mater. 23, 610–620 (2011).

[b48] Guo J., Ohkita H., Benten H. & Ito S. Near-IR femtosecond transient absorption spectroscopy of ultrafast polaron and triplet exciton formation in polythiophene films with different regioregularities. J. Am. Chem. Soc. 131, 16869–16880 (2009). 19886624 10.1021/ja906621a

[b49] Parkinson P., Mueller C., Stingelin N., Johnston M. B. & Herz L. M. Role of Ultrafast Torsional Relaxation in the Emission from Polythiophene Aggregates. J. Phys. Chem. Lett. 1, 2788–2792 (2010).

[b50] Ruseckas A. . Ultrafast photogeneration of inter-chain charge pairs in polythiophene films. Chem. Phys. Lett. 322, 136–142 (2000).

[b51] Lin H. . Fate of Excitations in Conjugated Polymers: Single-Molecule Spectroscopy Reveals Nonemissive “Dark” Regions in MEH-PPV Individual Chains. Nano Letters 9, 4456–4461 (2009). 19860455 10.1021/nl9027473

[b52] Luzhkov V. & Warshel A. Microscopic calculations of solvent effects on absorption spectra of conjugated molecules. J. Am. Chem. Soc. 113, 4491–4499 (1991).

[b53] Van Duijnen P. T. & De Vries A. H. Utopia dielectrica. Int. J. Quantum. Chem. 56, 523–531 (1995).

[b54] Lambert A., Yeguas V., Monard G. & Ruiz-López M. F. What is the effective dielectric constant in a β-cyclodextrin cavity? Insights from Molecular Dynamics simulations and QM/MM calculations. Comput. Theor. Chem 968, 71–76 (2011).

[b55] Warshel A. & Karplus M. Calculation of ground and excited-state potential surfaces of conjugated molecules .1. formulation and parametrization. J. Am. Chem. Soc. 94, 5612–5625 (1972).

[b56] Pariser R. & Parr R. G. A semi-empirical theory of the electronic spectra and electronic structure of complex unsaturated molecules .1. J. Chem. Phys. 21, 466–471 (1953).

[b57] Pariser R. & Parr R. G. A semi-empirical theory of the electronic spectra and electronic structure of complex unsaturated molecules .2. J. Chem. Phys. 21, 767–776 (1953).

[b58] Pople J. A. Electron interaction in unsaturated hydrocarbons. T. Faraday. Soc 49, 1375–1385 (1953).

[b59] Lobaugh J. & Rossky P. J. Computer simulation of the excited state dynamics of betaine-30 in acetonitrile. J. Phys. Chem. A 103, 9432–9447 (1999).

[b60] Lobaugh J. & Rossky P. J. Solvent and intramolecular effects on the absorption spectrum of betaine-30. J. Phys. Chem. A 104, 899–907 (2000).

[b61] Sterpone F. & Rossky P. J. Molecular modeling and simulation of conjugated polymer oligomers: ground and excited state chain dynamics of PPV in the gas phase. J. Phys. Chem. B 112, 4983–4993 (2008). 18380505 10.1021/jp711848q

[b62] Bedard-Hearn M. J., Sterpone F. & Rossky P. J. Nonadiabatic simulations of exciton dissociation in poly-p-phenylenevinylene oligomers. J. Phys. Chem. A 114, 7661–7670 (2010). 20597491 10.1021/jp103446z

[b63] Jailaubekov A. E. . Hot charge-transfer excitons set the time limit for charge separation at donor/acceptor interfaces in organic photovoltaics. Nat. Mater. 12, 66–73 (2013). 23223125 10.1038/nmat3500

[b64] Loewe R. S., Ewbank P. C., Liu J. S., Zhai L. & McCullough R. D. Regioregular, head-to-tail coupled poly(3-alkylthiophenes) made easy by the GRIM method: Investigation of the reaction and the origin of regioselectivity. Macromolecules 34, 4324–4333 (2001).

[b65] Hu Z. . Effect of the side-chain-distribution density on the single-conjugated-polymer-chain conformation. Chem.Phys.Chem. 14, 4143–4148 (2013). 24243782 10.1002/cphc.201300751

[b66] Adachi T. . Highly ordered single conjugated polymer chain rod morphologies. J. Phys. Chem. C 114, 20896 (2010).

