# Supplementary material for: An insight into non-emissive excited states in conjugated polymers
Source: Nat Commun. 2015 Sep 22;6:8246. doi: 10.1038/ncomms9246 (PMC4595598; doi:10.1038/ncomms9246)
Supplement: Supplementary Information — Supplementary Figures 1-7, Supplementary Tables 1-2, Supplementary Notes 1-3, Supplementary Method and Supplementary References [file ncomms9246-s1.pdf]

Supplementary Figures

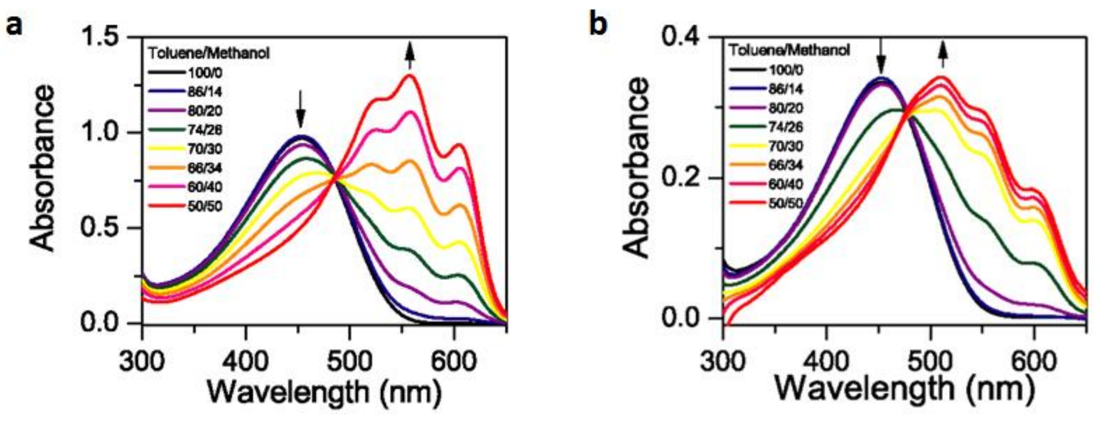

Supplementary Figure 1 | Absorption spectra of (**a**) triblock P3HT-b-PtBA-b-P3HT and (**b**) rr-P3HT (10 kDa) homopolymer upon going from toluene to toluene/methanol mixture gradually. The inset shows the volume ratio of toluene to methanol. As shown, the aggregates formed by the triblock exhibit more pronounced vibronic structures, indicative of better aggregation of P3HT.

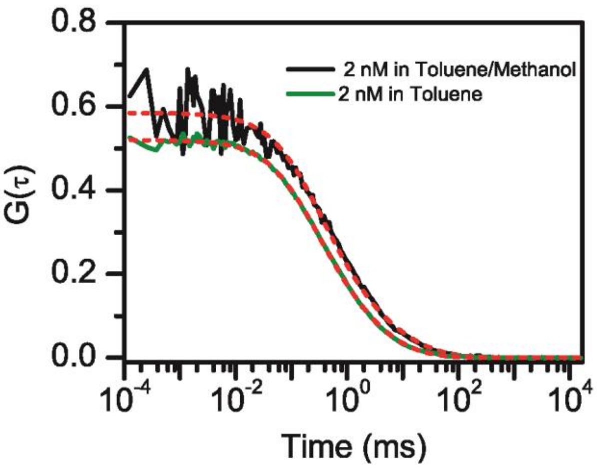

Supplementary Figure 2 | FCS correlation curves (solid lines) and corresponding fittings (red dashed lines) for triblock in toluene (green) and toluene/methanol (black) at same initial triblock concentration of 2 nM.

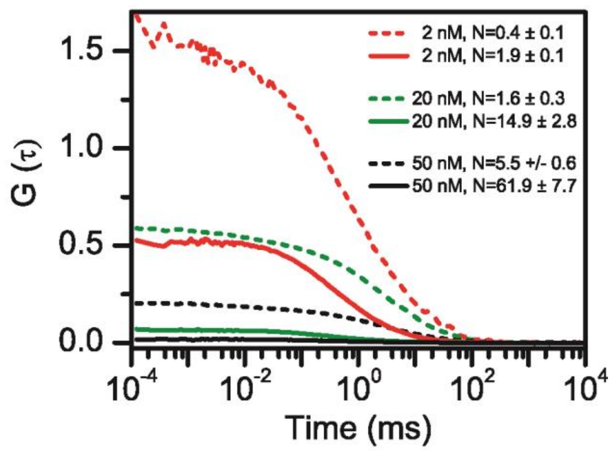

Supplementary Figure 3 | FCS correlation curves for triblock in toluene (solid lines) and toluene/hexane (dashed lines) at three same initial triblock concentrations. The inset presents the concentration and the number of emitters after background correction. As observed, in these studied concentrations, the aggregates in toluene/hexane have ~ 5-10 triblock molecules, a result similar to the case of aggregates in toluene/methanol.

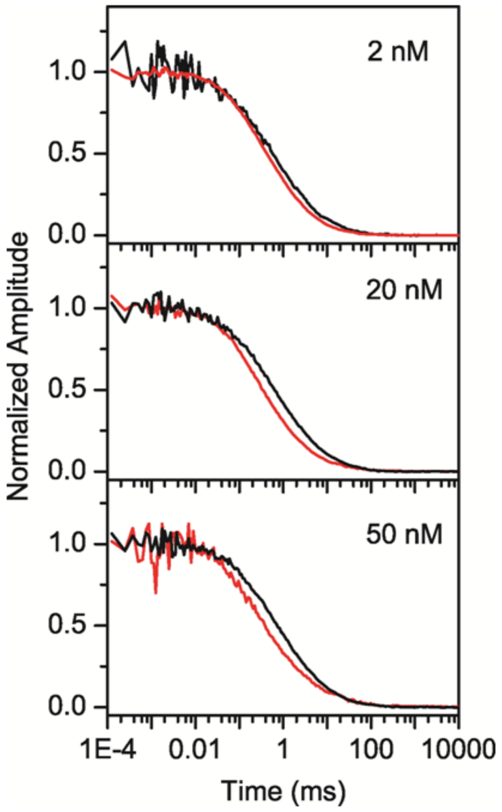

		solvent: toluene	solvent: toluene/methanol
τ_D (ms)	2 nM	11.9±0.6	37±15
	20 nM	16.6±1.5	40.4±8.1
	50 nM	16.0±4.9	44.0±8.0

Supplementary Figure 4 | FCS curves shown in Figure 3 in the main text for triblock in toluene (red) and toluene/methanol (50/50 vol.%) (black) at different concentrations. For comparison of diffusion time, the curves were normalized with G(0), which is the amplitude at correlation time τ=0 obtained from fitting (see Supplementary Discussion 1 for the fitting details). The table on the right summarizes the diffusion time (τ_D) obtained from fitting the curves shown on the left figure.

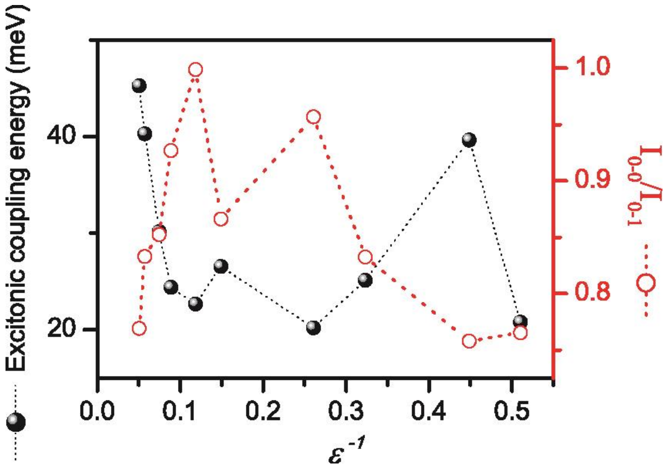

Supplementary Figure 5 | Excitonic coupling energy extracted from absorption spectra of pure aggregates (filled black circle) and $I_{0\text{-}0}/I_{0\text{-}1}$ emission intensity ratio (empty red circle) of pure aggregates in different solvent media. From left to right, the bad solvents become more and more nonpolar, i.e., from acetonitrile down to hexane, as listed in Table 1. The Huang-Rhys factor S and the main intramolecular vibrational energy ω_0 were calculated to be 1 and 0.18 eV, respectively, from a Franck-Condon analysis for the fluorescence spectra of triblock in toluene. Therefore, the expression for extracting excitonic coupling energy J_0 becomes the following:[1]

$$\frac{A_{0-0}}{A_{0-1}} = \frac{n_{0-0}}{n_{0-1}} \frac{(1-\frac{J}{\omega_0}e^{-s}\sum_{v>0}\frac{S^v}{v!v})^2}{(1-\frac{J}{\omega_0}e^{-s}\sum_{v\neq 1}\frac{S^v}{v!(v-1)})^2} \approx \left(\frac{1-0.96J_0/0.18}{1+0.294J_0/0.18}\right)^2.$$

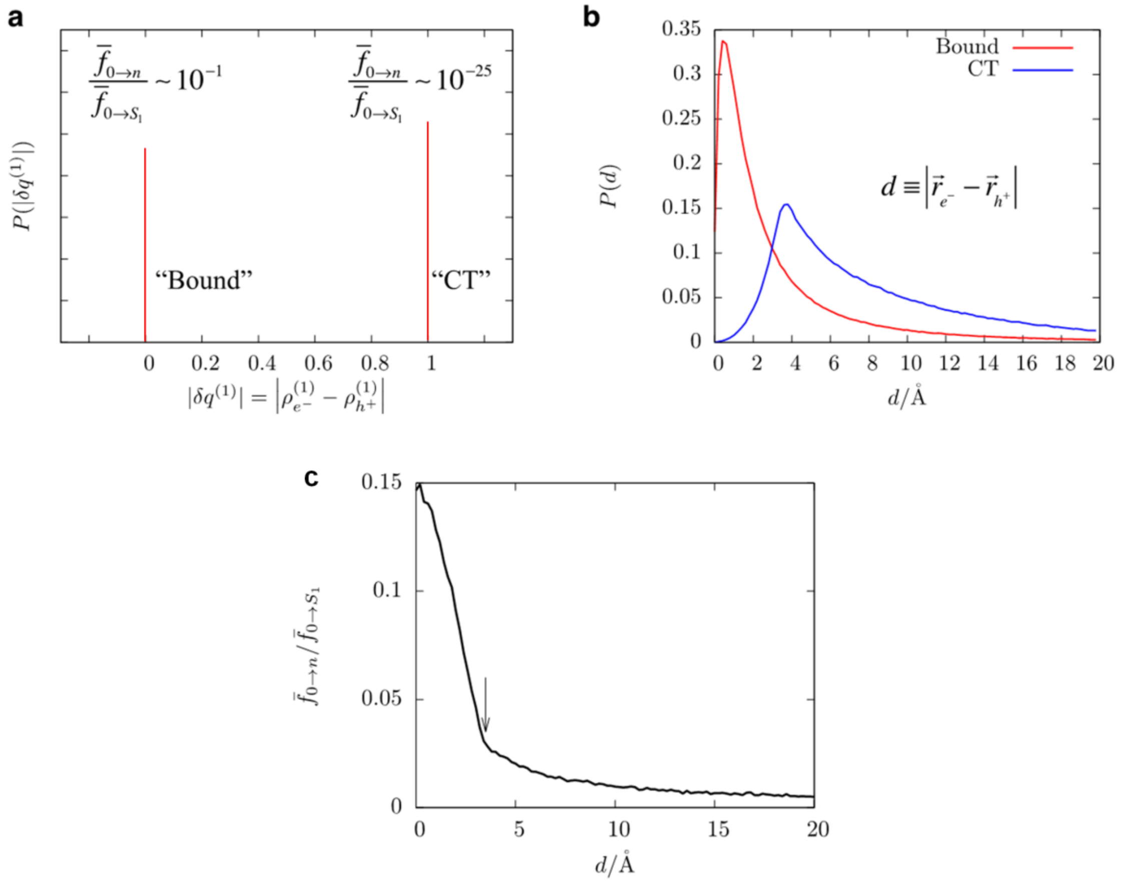

Supplementary Figure 6 | (**a**) The probability that the nth excited state (n<50) has a given value of $|\delta q^{(1)}|$ (i.e., the excess charge on one of the molecules). For each population we have computed the average oscillator strength (denoted as $\bar{f}_{0\to n}$) relative to that of the average optically bright state (denoted as $\bar{f}_{0\to S_1}$). (**b**) The probability that a member of the "Bound" or "CT" populations (as indicated in Panel a) have a given electron hole separation, $d \equiv |\vec{r}_{\mathrm{e}^-} - \vec{r}_{\mathrm{h}^+}|$, where $\vec{r}_{\mathrm{e}^-}$ and $\vec{r}_{\mathrm{h}^+}$ indicate the center of charge of the excited electron and corresponding hole wavefunction respectively. (**c**) The average oscillator strength, $\bar{f}_{0\to n}$, relative to that of the average optically bright state, $\bar{f}_{0\to S_1}$. The arrow indicates the position of the distance cutoff, $d = 3.5$ Å, used to distinguish the more strongly light-emitting exciton from weakly light-emitting polaron states.

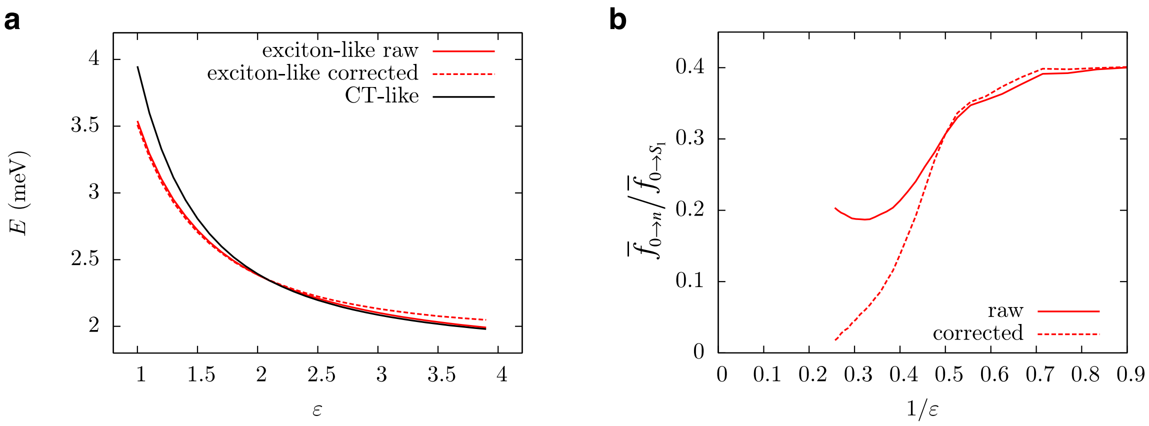

Supplementary Figure 7 | (a) A plot of the average energy of the lowest CT-type and exciton-type states. The plot contains two sets of exciton-type data. The data labeled 'raw' (solid red) is that which was generated explicitly with simulations (see Fig. 8a). The data labeled 'corrected' (dashed red) represents the raw data plus a linear shift. (**b**) A plot of the average oscillator strength, relative to that of the average optically bright state, for the 'raw' and 'corrected' data described in Panel (**a**).

Supplementary Tables

Supplementary Table 1 | Force field parameters. Units of energy, length, and angle are given in kcal/mol, angstrom, and radians respectively.

**Bond**	D_b	α	b_0	$K_b/2$
C-C	87.94	1.786	1.47	
C-S	65.00	1.700	1.78	
C-H	103.10		1.08	339.00
**Bond Angle**	$K_\theta/2$	θ_0	$F/2$	q_0
C-C-C	52.8	2.094	32.05	2.56
C-C-S	50.0	2.094	30.40	2.90
C-S-C	50.0	2.094	30.40	2.50
C-C-H	24.0	2.094	29.535	2.178
S-C-H	15.0	2.094	26.50	2.18
**Torsion Angle**	$K_\phi^{(1)}/2$	$K_\phi^{(2)}/2$	$K_{\theta\theta}$	
X-C-C-X	2.3	2.54	-6	
X-C-S-X	2.3	4.50	-10	
X-C-H-X		0.8		
**Nonbonded**	A	μ	B	
C****C	11392.83	3.0115	560.44	
S****S	30923.41	3.0115	1521.19	
H****H	4882.64	4.34	26.59	
C****S	18769.80	3.0115	923.33	
C****H	7458.36	3.56	134.97	
S****H	12287.72	3.56	222.36	

The PPP Hamiltonian used to describe the electronic structure of the pi-electron system is described in detail in Refs. [2-7]. The specific values of the parameter used in this study are shown in Supplementary Table 2.

Supplementary Table 2 | PPP parameters, in units of eV.

Site	$\gamma_{\mu\mu}$	α_μ
C	10.599	-11.45
S	9.7907	-20.00

Supplementary Notes

Supplementary Note 1

FCS curve fitting and correlation amplitude correction

The FCS curves were fitted with the following equation[8]:

$$G(\tau) = G(0)(1+\frac{\tau}{\tau_D})^{-1}\,(1+\left(\frac{s}{\mu}\right)^2\frac{\tau}{\tau_D})^{-1/2},$$

where, $G(\tau)$ is the correlation function of fluorescence fluctuations, $G(0)$ is autocorrelation amplitude at correlation time $\tau = 0$, τ_D is the diffusion time, and s and u is the radius and half-length of the observed volume, respectively.

The fitted correlation amplitude is 0.52 and 0.58 for the exemplary toluene and toluene/methanol samples shown in Supplementary Fig. 2, respectively. It should be noted that the measured amplitude should be corrected by a factor of $<F_{(t)}>^2/[<F_{(t)}>-<F_{BG}>]^2$, where $<F_{(t)}>$ and $<F_{BG}>$ are the time-averaged total fluorescence signal and background signal, respectively.[9] With the same excitation powder density (~ 6 μW), the $<F_{(t)}>$ for triblock in toluene and toluene/methanol is ~ 17.0 kHZ and 2.1 kHZ, respectively. And the background $<F_{BG}>$ is ~ 1.3 kHZ for both toluene and toluene/methanol. Therefore, for the toluene sample, the background corrected correlation amplitude $G(0)_{\rm corr}$ is $G(0)_{\rm corr} = G(0)(\frac{<F_{(t)}>}{<F_{(t)}>-<F_{(BG)}>})^2 = 0.52*(\frac{17}{17-1.3})^2 = 0.57$. So the correct number of emitters (N=1/*G*(0)) is 1.64. For the toluene/methanol sample, $G(0)_{\rm corr} = G(0)(\frac{<F_{(t)}>}{<F_{(t)}>-<F_{(BG)}>})^2 = 0.58*(\frac{2.1}{2.1-1.3})^2 = 3.0$, therefore the corrected number of emitters in 0.25. From toluene to toluene/methanol, the number of triblock polymer chains has been reduced from 1.64 to 0.25, meaning that about 6-7 chains have been assembled to make one aggregate.

Supplementary Note 2

Distinguish different characters of the excited states

Individual excited states were characterized as being exciton-type, polaron-type, or CT-type based on the following criteria. First, for each excited state the excess charge on molecule 1 due to the excitation, denoted as $\delta q^{(1)}$, was computed. Supplementary Fig. 6a illustrates that this gives rise to two distinct populations of states:

1. States for which $\left|\delta q^{(1)}\right| \approx 1$, meaning the excited electron and hole reside on opposite molecules. States in this population have a very small oscillator strength and are thus exceedingly weak light-emitters.
2. States for which $\left|\delta q^{(1)}\right| \approx 0$, meaning the excited electron and hole are distributed equally across both molecules. States in this population have a larger oscillator strength and electron-hole separations that are distributed around small values. For example see Supplementary Fig. 6b.

States in the former sub-population (i.e., $\left|\delta q^{(1)}\right| \approx 1$) are characterized as charge-transfer states. The latter sub-population (i.e., $\left|\delta q^{(1)}\right| \approx 0$) contains states with a broad range of electron-hole separations. States with larger electron-hole separations are polaron-like and, as illustrated in Supplementary Fig. 6c, are poorly light-emitting. Using the information plotted in Supplementary Fig. 6c we identified that there is a steep drop-off in oscillator strength for states with $d > 3.5$ Å. Since these states are not likely to contribute very much to the fluorescence quantum yield we use this observation as the basis to exclude such states from being characterized as ‘excitons’.

Supplementary Note 3

Sensitivity of fluorescence QY to simulation details

The quantitative behavior of the fluorescence quantum yield as a function of solvent dielectric is sensitive to simulation details. Supplementary Fig. 7 illustrates this sensitivity. Specifically we examine how the predicted fluorescence quantum yield responds to a small linear shift on the exciton energy levels. Such a shift is artificial but allows a systematic exploration of the consequences of slightly widening the energy gap between CT-like and exciton-like states. For the raw data the predicted fluorescence QY plotted in Supplementary Fig. 7b (compare to Fig. 5b in the main text) saturates to a value of approximately 20%. However, if the excited state energy levels include a subtle linear shift such as that shown in Supplementary Fig. 7a the fluorescence quantum yield is very similar to that seen experimentally. While we are not advocating that such a shift be applied to the data without justification, but make this comparison in order to highlight the sensitively. This suggests that in this regime even small computational inaccuracies can have large influence on details such as the particular value at which quenching is predicted to occur.

Supplementary Method

Theoretical simulation method

The potential energy function governing the configuration of the nuclei is given by,

$$V(r) = V_{\text{conj}}(r) + V_{\text{sat-conj}}(\text{r}),$$

where the subscript *conj* refers to the collection of atoms contributing to the conjugated *pi*-electronic system and the subscript *sat-conj* refers to the interactions between saturated and conjugated atoms. Following the notation of Ref. [10], the first term in the above equation is given by,

$$V_{\text{conj}}(r) = \sum_i D_b\left(e^{2\alpha(b_i-b_0)} - 2\,e^{\alpha(b_i-b_0)}\right) + \frac{1}{2}\sum_i [K_\theta(\theta_i-\theta_0)^2 + F(q_i-q_0)^2]$$
$$+\frac{1}{2}\sum_i\left(K_\phi^{(1)}\cos\phi_i + K_\phi^{(2)}\cos 2\phi_i\right) + \sum_i K_{\theta\theta}(\theta_i-\theta_0)^2\cos\phi_i$$
$$+\sum_{ij}\left(Ae^{-\mu r_{ij}} - Br_{ij}^{-6}\right),$$

where the first, second, third, fourth and fifth summation are taken over bonds, bond angles, torsional angles, and non-bonded pairs of atoms respectively. The quantities b_i, θ_i, q_i, ϕ_i, and r_{ij} denote bond length, bond angle between three consecutively bonded atoms, distance between first and third of three consecutively bonded atoms, torsional angle, and distance between atoms i and j respectively. The second term in the first equation is given by,

$$V_{\text{sat-conj}}(r) = \frac{1}{2}\sum_i (K_b(b_i-b_0)^2 + 2D_b) + \frac{1}{2}\sum_i [K_\theta(\theta_i-\theta_0)^2 + F(q_i-q_0)^2]$$
$$+\frac{1}{2}\sum_i\left(K_\phi^{(2)}\cos 2\phi_i\right) + \sum_{ij}\left(Ae^{-\mu r_{ij}} - Br_{ij}^{-6}\right).$$

Values of the force field parameters used in this study are included in Supplementary Table 1.

Supplementary References

1 Clark, J., Silva, C., Friend, R. H. & Spano, F. C. Role of intermolecular coupling in the photophysics of disordered organic semiconductors: aggregate emission in regioregular polythiophene. *Phys. Rev. Lett.* **98**, 206406, (2007).

2 Lobaugh, J. & Rossky, P. J. Computer simulation of the excited state dynamics of betaine-30 in acetonitrile. *J. Phys. Chem. A* **103**, 9432-9447, (1999).

3 Lobaugh, J. & Rossky, P. J. Solvent and intramolecular effects on the absorption spectrum of betaine-30. *J. Phys. Chem. A* **104**, 899-907, (2000).

4 Bedard-Hearn, M. J., Sterpone, F. & Rossky, P. J. Nonadiabatic simulations of exciton dissociation in poly-p-phenylenevinylene oligomers. *J. Phys. Chem. A* **114**, 7661-7670, (2010).

5 Sterpone, F., Bedard-Hearn, M. J. & Rossky, P. J. Nonadiabatic mixed quantum-classical dynamic simulation of pi-stacked oligophenylenevinylenes. *J. Phys. Chem. A* **113**, 3427-3430, (2009).

6 Sterpone, F. & Rossky, P. J. Molecular modeling and simulation of conjugated polymer oligomers: ground and excited state chain dynamics of PPV in the gas phase. *J. Phys. Chem. B* **112**, 4983-4993, (2008).

7 Jailaubekov, A. E. *et al.* Hot charge-transfer excitons set the time limit for charge separation at donor/acceptor interfaces in organic photovoltaics. *Nat. Mater.* **12**, 66-73, (2013).

8 Lakowicz, J. R. *Principles of Fluorescence Spectroscopy*. 3rd edn, (Springer, 2006).

9 Koppel, D. E. Statistical accuracy in fluorescence correlation spectroscopy. *Phys. Rev. A* **10**, 1938-1945, (1974).

10 Warshel, A. & Karplus, M. Calculation of ground and excited-state potential surfaces of conjugated molecules .1. formulation and parametrization. *J. Am. Chem. Soc.* **94**, 5612-5625, (1972).
